# Optimization of xylanase from *Pseudomonas mohnii* isolated from Simlipal Biosphere Reserve, Odisha, using response surface methodology

**DOI:** 10.1186/s43141-020-00099-7

**Published:** 2020-12-11

**Authors:** Manish Paul, Dipti Pravamayee Nayak, Hrudayanath Thatoi

**Affiliations:** Department of Biotechnology, North Orissa University, Takatpur, Baripada, Odisha 757003 India

**Keywords:** Lignocellulosic biomass, Xylanase production, Corn cob xylan, Xylanase, *Pseudomonas mohnii*, Response surface methodology

## Abstract

**Background:**

Xylanase has long been recognized as a widely used industrially important enzyme. There are some bacterial species already reported to produce xylanase which have potent xylanolytic activity towards the use of this enzyme in the production of bioethanol from lignocellulosic biomass. In this view, an efficient xylanolytic bacterial strain was isolated and screened from the soil sample of Simlipal Biosphere Reserve. Enzymatic assay for the xylanase activity was evidenced from the most potent bacterial strain, and the culture condition was optimized for obtaining the maximum enzyme activity. The most potent xylanolytic strain was also identified using biochemical and molecular methods.

**Results:**

Nineteen xylanolytic bacteria (SXB1-SXB19) were isolated from Simlipal forest soil samples following dilution plate technique using corn cob xylan-enriched nutrient agar medium and screened for their xylanase-producing ability. Among these isolates, SXB19 showed maximum xylanolytic potential with a halozone size of 2.5 cm as evident in the formation of prominent yellow patches surrounding its growth in xylan-enriched nutrient agar plate. In unoptimized condition, SXB19 showed the highest enzymatic activity of 22.5 IU/ml among the 19 bacterial strains. In order to optimize the culture conditions for maximizing the xylanase production, Box-Behnken design of response surface methodology (RSM) was used. Four variables such as incubation time, pH, substrate (corn cob xylan) concentration, and temperature were considered for the RSM optimization study. From the results, it is evident that in an optimized condition of incubation time 36 h, pH 6.0, xylan concentration 0.5%, and temperature 42.5 °C, the enzyme activity reached a maximum of 152 IU/ml with nearly 6.75 times increase from the unoptimised condition. Besides, xylanase production from SXB19 was considerable in the presence of xylan followed by starch, nitrogen source such as urea followed by yeast extract, and mineral ion sources such as KCl followed by MgSO_4_ and ZnSO_4_. From different biochemical tests, 16S rRNA gene sequencing, and phylogenetic analysis, the bacterial strain SXB19 was identified as *Pseudomonas mohnii*.

**Conclusion:**

The isolation of *Pseudomonas mohnii*, a potent xylanolytic bacterium from Simlipal, is a new report which opens up an opportunity for industrial production of xylanase for bioethanol production and other applications.

**Graphical abstract:**

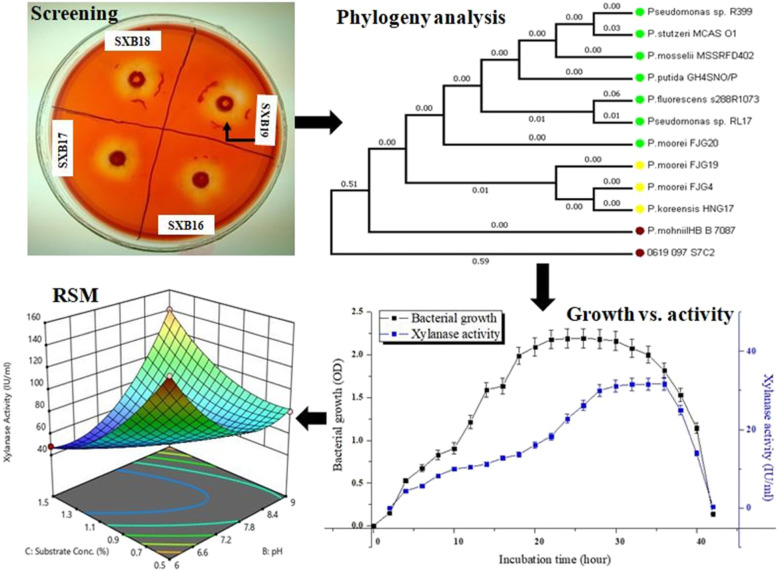

**Supplementary Information:**

The online version contains supplementary material available at 10.1186/s43141-020-00099-7.

## Background

In recent years, there is a tremendous pressure on world fossil fuel supply and reserve [1]. The demand for fossil fuel is increasing day by day, and the available resources are rapidly decreasing indicating that soon, it will be disappeared [2]. The International Energy Agency (IEA) predicts that the world’s energy demand will increase by more than 55% between 2005 and 2030, and oil demand is projected to grow from some 85 million barrels per day currently to some 120 million barrels per day by 2030 [3]. It is also known that the large-scale use of fossil fuels all over the world increase CO_2_ level and accumulate greenhouse gasses which make the environment polluted and unsustainable [2]. To maintain the sustainability of fuel demand and cleanliness of the environment, there is an urgent need of producing renewable and environment-friendly fuels [4].

In this context, biofuel seems to be an alternative to fossil fuel which has reduced impact on environment pollution [5]. The global production and use of biofuels like bioethanol and biodiesel as the alternative energy source have shown to significantly promote in recent years among which only bioethanol covers 85%.

Bioethanol can be produced from different biomass feedstocks containing fermentable sugars or complex carbohydrates through appropriate fermentation technology. These feedstocks can be classified into three groups, which are sugary crops, starchy crops, and lignocellulosic biomass [6]. The bioethanol production from sugary and starchy crops is known as the first-generation bioethanol while that of lignocellulosic biomass is considered as the second-generation bioethanol. First-generation biofuel production comprises food crops such as maize, rice, wheat, barley, potato, sugarcane, and vegetable oil, for example, soybean oil, sunflower oil, olive oil, canola oil, and mustard oil*.* The production of first-generation bioethanol is generally assumed to be one of the main causes of scarcity of food staff. Therefore, the recent research efforts have been focused on lignocellulosic biomasses, the second-generation bioethanol, as they are mostly waste materials, available at a low price, rich in carbohydrates, and noncompetitive with the food chain [7].

Presently, researchers are directed on developing multiple processes to produce biofuel energy from lignocellulosic biomass [8]. Lignocellulosic biomass consists of plant cell walls whose primary structural components are polysaccharides such as cellulose, hemicellulose, and heterogeneous polyphenolic substance like lignin. Among these three, cellulose and hemicellulose are converted to ethanol through fermentation whereas lignin is recalcitrant in nature and it actively inhibits fermentation. However, the components of lignocellulosic biomass vary substantially, according to plant species, soil fertility, fertilization practice, and climate. For agricultural residues such as rice straw, wheat, and corn stover, the plant cell wall was reported to contain about 40% cellulose, 30% hemicelluloses, and 15% lignin [9].

Cellulose is a homopolymer of hexose sugar glucose which is the main contributor for bioethanol production followed by hemicelluloses. Hemicellulose is a combination of different polysaccharides and is mainly composed of pentose sugars such as xylose and arabinose. This polysaccharide can be easily degraded into monosaccharides such as xylose, galactose, fructose, dextrose, arabinose, and mannose because of its low degree of polymerization [10–12]. Xylan is a polysaccharide and a major component of hemicelluloses in plants which is made of xylose molecules. Next to cellulose, xylan is the second most abundant renewable polysaccharide in nature and is responsible to hold the plant cell wall together. Complete hydrolysis of xylan to xylose needs a complex of xylanolytic enzymes such as endoxylanase, β-xylosidase, α-arabinofuranosidase, acetyl esterase, and α-glucuronidase. Since hemicullulose hydrolysis is an important step in bioethanol production, isolation and evaluation of hemicellulase enzyme-producing microorganisms have been an important field of research in this essence.

Xylanase (E.C.3.2.1.8) is the class of glycosyl hydrolases enzymes which hydrolyze hemicellulose by degrading the linear polysaccharide β-1,4 xylan to xylose [13]. Xylanase has a wide range of applications in biotechnology which include bio-pulping of wood, bleaching of pulp [14], processing of foods [15], and conversion of lignocellulosic substances into bioethanol [16]. In comparison with a chemical pretreatment, the microbial xylanase enzyme has a low environmental impact and hence more eco-friendly during the applications in the abovementioned industrial purposes [17]. Xylanase-mediated hydrolysis of lignocellulosic biomass reported to produce oligosaccharides which are further used as functional food additives or alternative sweeteners with beneficial properties [18]. One of the critically important enzymatic activities required for the depolymerization of xylans is endo-1,4-β-xylanase activity. These enzymes cleave the β-1,4 glycosidic linkage between xylose residues in the backbone of xylans present in lignocellulosic biomass [19]. Xylanase is produced by many microorganisms such as bacterial as well as fungal species. This enzyme plays a significant role in such microorganisms that live on plant sources for the degradation of plant materials into usable nutrients [20]. Hence, it is important to isolate and identify xylanase-producing microorganisms from the natural environment and evaluate their bioethanol production ability, an important field of biotechnology research. Forest soil can harbor a large number of lignocellulolytic microorganisms including xylanase-producing one due to the availability of huge quantities of lignocellulosic biomasses and can be explored for xylanolytic bacteria. Numerous physicochemical factors serve as the catalyst during the action of xylanase enzyme which in turn enhance the quality of enzyme and its mass production and substrate specificity as well even in extreme conditions such as high temperature, high or low pH, and high or low salinity [21]. Xylanase can be produced by two main methods, e.g., solid-state fermentation and submerged fermentation, where all the parameters can be controlled and manipulated for the purpose of mass production of the enzyme [22, 23].

Keeping these in view, the present study aims at isolating and screening an efficient xylanase-producing bacterium from the soil samples of Simlipal Biosphere Reserve (SBR), located in the northern part of Odisha, India, and to optimize the xylanase production ability of that bacterium using the response surface method for its possible use in bioethanol production from lignocellulosic biomasses.

## Methods

### Collection and storage of soil sample

Soil samples were collected from different locations from two forest sites viz. Sitakund and Lulung of Simlipal Biosphere Reserve (21° 10′ to 22° 12′ N latitude and 85° 58′ to 86° 42′ E longitude), located in Mayurbhanj district in Odisha, India. The reserve is a compact mass of natural forest spread over a total area of 5569 km^2^ with a core area of 845 km^2^ and a buffer zone of 2129 km^2^ comprising 16 forest ranges that surrounds a transitional zone of 2595 km^2^ [24]. About a 1-cm top layer of soil was removed during the collection of soil samples. The soil samples were packed in sterile polythene bags and kept in an ice box. Then, these samples were transported to the laboratory and stored at 4 °C for conducting further experiments.

### Isolation of xylanolytic bacteria

One gram of soil sample was measured aseptically and transferred to a 250-ml flask containing 100-ml sterile water and covered with an aluminum foil. The flask was shaken for 15–20 min in an orbital shaker to make the stock solution having a dilution of 10^−2^. One-milliliter suspension from stock solution was added in 9 ml of sterilized water taken in a sterile test tube, and suspension was then serially diluted to obtain 10^−5^ or 10^−6^ dilutions. The suspension with 10^−5^ or 10^−6^ dilutions was then used as inoculums for pour plating performed in a laminar air flow by using corn cob xylan-enriched (obtained from Sisco Research Laboratories Pvt. Ltd. (SRL), India) nutrient agar (g/100 ml: nutrient broth, 1.3 g; agar, 2 g) medium. Inoculated plates were incubated at a 37 °C temperature in a bacterial incubator for 48 h to obtain xylanolytic bacterial colonies.

### Screening of xylanolytic bacteria

Screening of xylanolytic activity of bacterial isolates was carried out in nutrient agar medium containing 0.5% of corn cob xylan. For this purpose, pure cultures of selected colonies were prepared through repeated streak plating, and a loopful of culture from the pure cultures was inoculated on nutrient broth xylan medium and broth cultures were incubated for 18 h. Ten microliters of each bacterial culture (intact bacterial cell in broth) was inoculated inside a 4-mm pore made on the xylan agar plates. Xylan agar plates containing bacterial inoculums were then incubated at 37 °C for 72 h. Plates were stained by 0.1% Congo red solution and kept for 1 h followed by destaining using 1 M NaCl for 15 min for determination of xylan hydrolyzing zone surrounding the bacterial growth on xylan agar plates [25]. After destaining, xylan hydrolyzing halo zone (*H*) and colony diameter (*C*) were measured for the individual bacterial strain to calculate hydrolysis index (*H*:*C*).

### Determination of bacterial xylanase activity

Xylanase activity of the isolated xylanolytic bacteria was determined by the DNS method [26]. Nineteen bacteria showing distinct hydrolyzing zone on xylan-enriched nutrient agar plates were selected as Simlipal xylanase bacteria and designated as SXB1 to SXB19. The amount of reducing sugar hydrolyzed by the xylanase activity of all the nineteen xylanolytic bacterial isolates (SXB1-SXB19) was measured which showed a xylan-hydrolyzing zone surrounding their growth. After an incubation of 24 h, freshly cultured bacterial broths of each xylanolytic bacterial isolates were individually transferred to a sterile 1.5-ml eppendorf tube. Each eppendorf tube was centrifuged at 10,000 rpm for 10 min at 4 °C. After centrifugation, the supernatant formed in each eppendorf tube was separated carefully using pipettes from the pellets and used to prepare the reaction mixture for enzyme assay. The substrate solution was prepared by adding 1% of corncob xylan with phosphate buffer (0.1 M) at pH 6.8. Substrate solution of 0.5 ml and each bacterial supernatant (crude enzyme) of 0.5 ml were taken in an assay tube to make 1- ml reaction mixture. Assay tubes containing the reaction mixture were then incubated at 55 °C for 15 min in a water bath. After the incubation, 1.5 ml of DNS solution was mixed to each assay tube and boiled at 100 °C for 10 min to stop the reaction. The amount of reducing sugar released in the hydrolysis by each bacterial supernatant was measured using a UV-VIS spectrophotometer (Systronic-119) at an absorbance value of 540 nm. One unit (IU) of xylanase activity was measured as 1 μMol of xylose liberated per milliliter of enzyme per minute. For the enzyme assay, the reaction control was prepared without a bacterial supernatant (crude enzyme) and was simultaneously run under the DNS protocol for comparison with other samples.

### Growth curve analysis

The growth curve was studied for the most efficient bacterial strain SXB19 that showed the highest xylanolytic activity. One loopful of pure culture was transferred into 100 ml of sterile xylan-enriched media in a conical flask and kept in an incubator. A control containing 100 ml xylan broth was also prepared without inoculating the bacterial culture and was analyzed simultaneously with the inoculated sample for comparison. The culture was taken at regular 2-h intervals for 42 h to determine the bacterial growth by measuring the absorbance at 600 nm using a UV-Vis spectrophotometer. A growth curve of cell concentration against time was plotted using the obtained absorbance values. Xylanase activity was also measured at 2 h intervals during bacterial growth using the DNS method as described above.

### Phenotypic characterization of xylanase-degrading bacteria

For phenotypic identification, morphological and biochemical studies were undertaken. To study the morphology, Gram’s staining was carried out for the xylanolytic bacterium SXB19. After Gram’s staining, the bacterium was observed under a phase-contrast microscope (× 100 objectives) to determine its shape, size, and response to Gram stain (Gram positive or Gram negative).

### Biochemical characterization of xylanolytic bacteria

The bacterial strain SXB19 was further subjected to biochemical tests as per *Bergey’s Manual of Systematic Bacteriology* [27]. These tests are useful for the identification of the unknown bacterium. A number of biochemical tests such as catalase test, oxidase test, urease test, methyl red test, Voges-Prausker test, citrate utilization test, carbohydrate metabolism test, oxidation/fermentation test, indole test, hydrogen sulfide (H_2_S) test, gelatin hydrolysis test, and coagulase test were performed.

### Molecular identification of xylanolytic bacteria

Molecular identification of the most potent xylanolytic strain SXB19 was done by 16S rRNA gene sequencing. DNA of the SXB19 bacterial strain was extracted using standard methods [28]. The 16S rRNA gene was amplified by PCR using universal primers 27F (5′-AGAGTTTGATCCTGGCTCAG-3′) and 1492R (5′-GGTTACCTTGTTACGACTT-3′). Amplified 16S rRNA gene was then purified using a Gel DNA extraction kit (Qiagen, Seoul, South Korea) and sent for sequencing to Applied Bioscience Eurofins, Bangalore.

### Construction of phylogenetic tree

Using the forward and reverse sequence data of the bacterium SXB19 obtained from 16S rRNA gene sequencing, a consensus 16S rRNA gene sequence of the bacterium was generated by the CAP3 web server [29]. Other homolog 16S rRNA gene sequences were compared with the consensus sequence generated for the SXB19 isolate using the BLASTN tool (https://blast.ncbi.nlm.nih.gov/Blast.cgi) available at the GenBank database of the NCBI server. The closely related homolog sequences were considered for both the pair-wise and multiple sequence alignment. Pair-wise and multiple sequence alignment using the ClustalW tool were performed, and the output of alignment was used for the construction of phylogenetic tree in the MEGA 7.0.26 (Molecular Evolutionary Genetics Analysis) software using the neighbor-joining (NJ) [30].

### Evaluation of carbon, nitrogen, and metal sources in xylanase production by SXB19 bacterium

To select the preferable carbon and nitrogen sources to enhance the production of xylanase enzyme by the bacterium SXB19, the bacterium was grown separately in a medium supplemented with different carbon sources such as glucose, sucrose, fructose, starch, and nitrogen sources such as yeast extract, urea, sodium nitrate, ammonium nitrate, and peptone in a concentration of 0.4% (w/v). Further, metal ion sources such as CoCl_2_, CaCl_2_, MgSO_4_, MnSO_4_, CuSO_4_, FeSO_4_, KCl, and ZnSO_4_ were also added individually in a concentration of 0.1% w/v to the bacterial growth medium to determine their role on enzyme activity [31]. To assess the effect of various carbon sources on enzyme production, corncob xylan in the basal medium was replaced with other carbon sources, such as glucose, fructose, sucrose, and starch. In addition, corncob xylan was also tested as a carbon source as control. For the determination of the role of different nitrogen sources on enzyme production, tryptone and peptone in the basal medium were replaced with yeast extract, urea, sodium nitrate, and ammonium nitrate. In case of the determination of the effect of metal ion sources on enzyme production, K_2_HPO_4_ and NaCl were supplemented with other metal ion sources that have been mentioned earlier.

### Optimization of xylanase enzyme production

#### Experimental design and statistical optimization of xylanase production using RSM

A Box-Behnken design was used to investigate the combined effect of four variables such as incubation time, pH, xylan concentration, and temperature on xylanase enzyme production. Different degrees of the variables (A, B, C, and D) at three levels (+ 1, 0, − 1) were used in the experimental design and are presented in Table 1. The independent variables were coded − 1 and + 1 as low and high levels, respectively [32]. The Box-Behnken design is most widely used for the preparation of quadratic response surfaces and a second-degree polynomial model to analyze the pattern of enzyme production. This second-degree polynomial model is used for process optimization by carrying out a set of experimental runs [33]. Box-Behnken designs are used to generate higher-order response surfaces using fewer required runs than a normal factorial technique. This technique essentially suppresses selected runs in an attempt to maintain the higher-order surface definition. The Design-Expert (version 12, Stat-Ease Inc., Minneapolis, USA) statistical software was used to develop the experimental design for optimizing the growth condition to obtain maximum xylanase activity. The experimental design is set up on a number of runs (*N*) according to the following equation: *N* = *k*^2^ + *k* + *C*_*p*_, where *k* and *C*_*p*_ are the factor number and replications number which are 4 and 3, respectively, in the present study. The predicted model using the second-degree polynomial equation resulted in 27 different experimental runs in the current work. The second-degree polynomial equation for the predicted model can be represented as follows:

$$ Y={b}_0+{b}_1\mathrm{A}+{b}_2\mathrm{B}+{b}_3\mathrm{C}+{b}_4\mathrm{D}+{b}_{12}\mathrm{AB}+{b}_{13}\mathrm{AC}+{b}_{23}\mathrm{BC}+{b}_{24}\mathrm{BD}+{b}_{11}{\mathrm{A}}^2+{b}_{22}{\mathrm{B}}^2+{\mathrm{b}}_{33}{\mathrm{C}}^2+{\mathrm{b}}_{44}{\mathrm{D}}^2 $$

**Table 1:** Actual levels for the four variables Box-Behnken design in response surface methodology of the xylanase enzyme extracted from the bacterium *Pseudomonas mohnii*

Independent variables	symbols	Coded and actual levels		
		−1(low)	0	+ 1(high)
**Incubation time (h)**	A	12	36	60
**pH**	B	6.0	7.5	9.0
**Xylan concentration (%)**	C	0.5	1	1.5
**Temperature (°C)**	D	35.0	42.5	50.0

In this equation, *Y* is the predicted response in terms of xylanase activity. In this study, the Box-Behnken design consisted of four factors viz. incubation time (A) [12, 36, and 60 h], pH (B) [6, 7.5, and 9.0], substrate concentration (C) [0.5, 1, 1.5 (%)], and temperature (D) [35, 42.5, and 50 (°C)] further production of xylanase enzyme from SXB19 strain of xylanolytic bacteria. *b*_0_ is the inter shape or offset term taken from the equation; *b*_1_, *b*_2_, *b*_3_, and *b*_4_ are the linear effect; and *b*_11_, *b*_22_, *b*_33_, and *b*_44_ are quadratic terms. Further, the RSM data were undertaken for regression analysis and analysis of variance (ANOVA) to fit the model according to abovementioned equation. Adequacies of the resulted model were validated by regression analysis and *R*^2^ analysis. *F* value was verified to determine the statistical probability of all calculated models at a 5% level of significance [33, 34]. To understand the relationship between predicted response and experimental level of each factor and to explore the optimum xylanase production condition, the fitted equation was expressed as optimized three-dimensional surface plots, which is also prepared using the software Design-Expert.

#### Culture of bacteria according to the experimental condition designed by RSM

For the bacterial culture, growth media containing (g/100 ml) 1.7 g tryptone, 0.3 g peptone, 0.25 g K_2_HPO_4_, and 1.8 g NaCl were prepared and the pH of the media adjusted to three different values (6.0, 7.5, and 9) according to the conditions designed by RSM. The growth media were supplemented with different concentrations of xylan (0.5, 1, and 1.5%) as mentioned in the experimental design. All the flasks containing culture broth were autoclaved at 121 °C for 15 min. After autoclave, the flasks were set to cool at room temperature and placed under UV laminar flow for sterilization. After few minutes, each flask was inoculated with 1% of standard inoculum (v/v) of the SXB19 xylanolytic bacterial strain which was freshly cultivated for 24 h. The inoculated flask was then incubated on a rotary shaker (120 rpm) at a specific temperature (35, 42.5, 50 °C) and for a specific time (12, 36, 60 h) as given in the experimental design from the RSM calculation.

#### Estimation of xylanase production

After the incubation according to the designed experimental conditions, 1 ml of bacterial culture from each flask was taken in the Eppendorf tube. In this study, xylanase activity was measured in duplicate by preparing two Eppendorf tubes containing bacterial culture for each experimental condition. Each Eppendorf tube containing 1 ml bacterial culture broth was centrifuged at 10,000 rpm for 10 min at 4 °C. After centrifugation, the bacterial supernatants were collected to perform the DNS assay for the determination of xylanase activity [26]. The predicated values of xylanase activity obtained from each experimental condition were then used to generate three-dimensional surface plots, regression analysis, and analysis of variance (ANOVA).

## Results

### Isolation and screening of xylanolytic bacteria

**Fig. 1:**
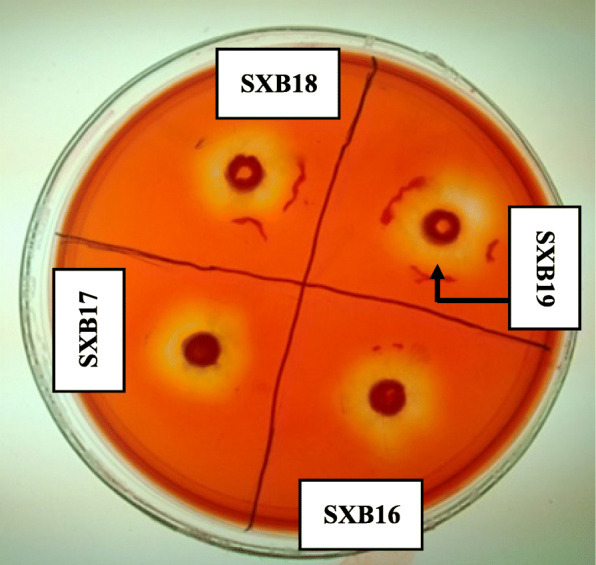
Clearing zone generated by xylanolytic bacteria SXB19 in xylan agar plates after Congo red staining

A total of 19 xylanolytic bacteria were isolated from soil samples from Simlipal Biosphere Reserve, Odisha, using a xylan-rich medium and designated as Simlipal xylanolytic bacteria (SXB1-SXB19). These bacteria were purified by subsequent sub-culturing and used for further experiments. Comparative xylan-hydrolyzing zone has been determined for all the isolated bacteria. The range of the average diameter of xylan-degrading halo size (*H*) observed for all the strains was between 0.6 and 2.5 cm (Table 2). Among the different bacterial isolates, SXB19 showed a comparatively higher halozone formation in xylan-enriched nutrient agar plate. The strain SXB19 exhibited the most prominent yellow patch surrounding its growth among all the xylan-degrading bacteria with a halozone size of 2.5 cm implicating the most efficient xylan-degrading bacterial strain (Fig. 1). The average colony diameter (*C*) for all the bacterial isolates was the same with a value of 0.567 cm. After comparing the ratio of the halo’s diameter and colony diameter (*H*:*C*), it was also found that the strain SXB19 has the highest *H*:*C* value of 4.40 cm among all.

**Table 2:** Xylan degrading activity of different isolated bacterial strains based on the measurement of xylan hydrolyzing zone

Sl No.	Isolate name	Halo’s diameter horizontally X(in cm)	Halo’s diameter vertically Y(in cm)	Average halo’s diameter [H] =(X + Y)/2 (in cm)	Colony diameter [C]	H:C
**1**	**SXB1**	0.7	0.5	0.6	0.567	1.06
**2**	**SXB2**	1	1	1	0.567	1.76
**3**	**SXB3**	1.1	1.1	1.1	0.567	1.94
**4**	**SXB4**	1	1.1	1.05	0.567	1.85
**5**	**SXB5**	0.9	0.8	0.85	0.567	1.49
**6**	**SXB6**	1.39	1.29	1.34	0.567	2.36
**7**	**SXB7**	1.1	1.6	1.35	0.567	2.38
**8**	**SXB8**	1.39	1.2	1.295	0.567	2.28
**9**	**SXB9**	2	2.2	2.1	0.567	3.70
**10**	**SXB10**	2	2	2	0.567	3.53
**11**	**SXB11**	1.95	2.1	2.025	0.567	3.57
**12**	**SXB12**	2.12	2.13	2.125	0.567	3.75
**13**	**SXB13**	2.1	2	2.05	0.567	3.61
**14**	**SXB14**	2.14	2.12	2.13	0.567	3.76
**15**	**SXB15**	1.9	1.8	1.85	0.567	3.26
**16**	**SXB16**	2.2	2.2	2.2	0.567	3.88
**17**	**SXB17**	1.9	1.7	1.8	0.567	3.17
**18**	**SXB18**	2.3	2.45	2.375	0.567	4.19
**19**	**SXB19**	2.5	2.5	2.5	0.567	4.40

### Determination of bacterial xylanase activity

**Fig. 2:**
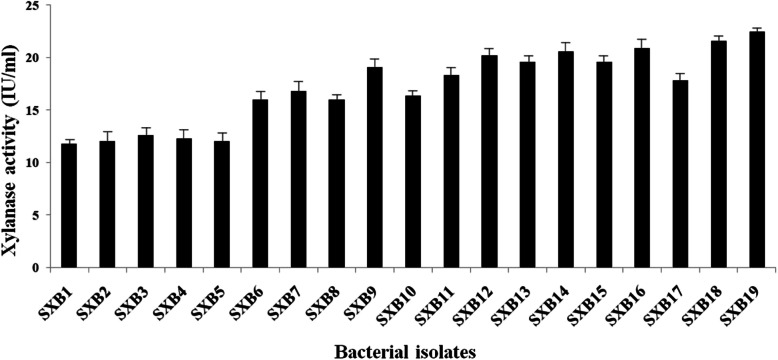
Xylanase activity of bacteria using dinitrosalicyclic acid (DNS) method

The result of bacterial xylanase activity determination is given in Fig. 2. In accordance with the qualitative estimation performed during screening, the bacterial isolate SXB19 showed the highest xylanase activity as measured in the DNS method. The obtained high spectrometric absorbance values indicated the formation of maximum amount of reducing sugar after the catalytic activity of xylanase on the substrate xylan. According to the obtained result, xylanase activity of the bacterial strain SXB19 was recorded to have the highest enzymatic activity of 22.5 IU/ml after a 24-h incubation time.

### Growth curve analysis

**Fig. 3:**
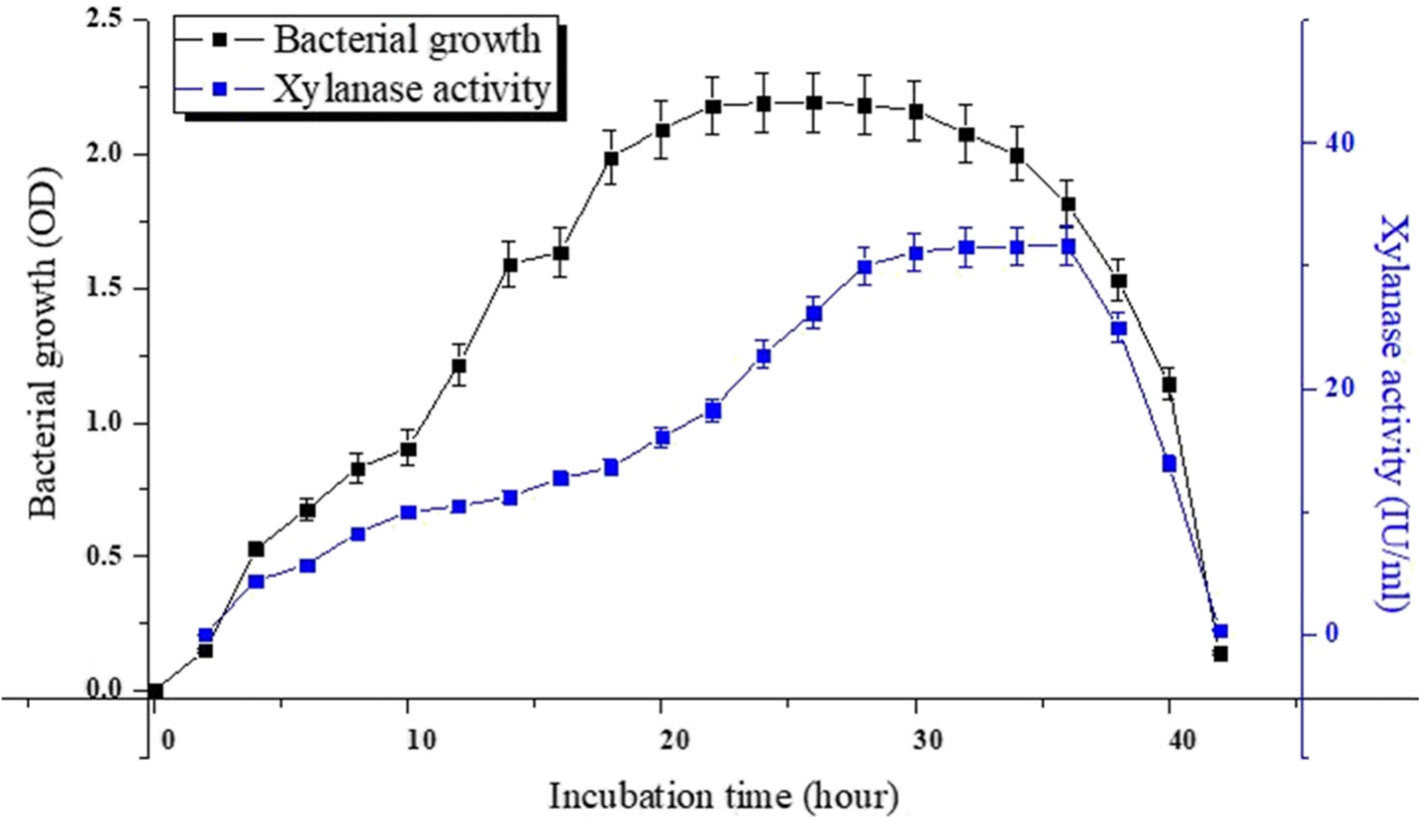
Bacterial growth curve and xylanase activity of isolated xylanolytic bacteria SXB19. The bacterium was cultured in a xylan-enriched nutrient broth medium containing 0.5% of corn cob xylan and was incubated at 37 °C

Figure 3 shows the growth profile of bacterial isolate SXB19 along with its xylanase activity in xylan-enriched nutrient broth. The lag phase of the bacterium was recorded up to the first 2 h after the inoculation. It is found from the experiment that the exponential phase of xylanolytic bacteria SXB19 started after 2 h which extends up to 22 h. Thereafter, the growth of the bacteria enters into the stationary phase. The average absorbance value of bacterial growth for SXB19 at its stationary phase was recorded 2.182, and the maximum values reached 2.193 in 26 h of incubation. Xylanase activity of SXB19 culture supernatant was detected after 2 h of the bacterial growth started. SXB19 showed a gradual increase in xylanase activity between 4 and 18 h of its exponential growth phase with a value of 5.39 to 13.62 IU/ml. Further, in the present study, during 20 to 28 h of SXB19 growth, the enzymatic activity increased rapidly with a value of 16.13 to 30 IU/ml. Xylanase activity of SXB19 remained more or less constant within the time period from 30 to 36 h. The highest xylanase activity of 31.7 IU/ml was observed at 36 h. In the dye off phase of the bacterial strain, the absorbance value suddenly dropped from 2.0 at 34 h to 1.53 at 38 h which finally reached to 0.142 at the end of 42 h. Xylanase activity is also reported to be significantly dropped down from 25 to 0.36 IU/ml during the time period of the dye off phase.

### Morphological and biochemical characterization of xylanolytic bacteria

For morphological characterization, the xylanolytic strain SXB19 was studied for its shape and the pattern of gram staining. The strain SXB19 was found to be a rod-shaped Gram-negative bacterium. Further biochemical characterizations were also performed to identify the xylanolytic bacterial isolate SXB19. The results of biochemical tests are presented in Table 3. In the catalase test, a positive result is indicated by the production of bubbles in the form of oxygen in the nutrient agar plates. The xylanolytic bacterial strain SXB19 showed a negative result towards the catalase test. In the oxidase test, the xylanolytic train SXB19 showed a positive reaction indicated by a change in color of the broth to colorless within few seconds. In the urease test, a positive result was indicated by the pinkish-red coloration of the medium after the inoculation with SXB19 strain. In the methyl red test, the SXB19 strain showed a positive result indicated by the change of color of the growth medium to red. The SXB19 strain showed a positive result in the Voges-Proskauer test indicated by the development of a crimson yellow color of the medium. In the citrate utilization test, the growth of SXB19 xylanolytic strain on the slants accompanied by a change of color of slants to blue indicates a positive result. In the carbohydrate metabolism test, a positive result by SXB19 strain is indicated by the change of the color of the broth to a yellow color along with gas production. The oxidative-fermentative test determined that the SXB19 bacterial strain was oxidative. The bacterial strain showed negative results for the indole, H_2_S, and coagulase tests. According to the observation from gelatin hydrolysis, this bacteria was shown to have a negative result towards this test. Based on these abovementioned characteristics observed from the morphological and biochemical tests and references from *Bergey’s Manual of Systematic Bacteriology*, the xylanolytic bacterial isolate SXB19 was tentatively identified as a member of the genus *Pseudomonas*.

**Table 3:** Morphological and biochemical characterization of SXB19 xylanolytic bacterial isolate

**Morphological character**	**Pattern**
Shape of the bacterial cell	Rod
Gram staining	Negative
**Biochemical tests**	**Response**
Catalase	**–**
Oxidase	**+**
Urease	**+**
Methyl red (MR)	**+**
Voges Proskauer (VP)	**+**
Citrate utilization	**+**
Carbohydrate metabolism	**+**
Oxidative/Fermentative	Oxidative
Indole	–
H_2_S	–
Gelatin hydrolysis	–
Coagulase	–

### Molecular identification of xylanolytic bacterial strain SXB19

In the 16S rRNA gene sequencing, a molecular approach was undertaken to identify the bacterium at its species level. The forward and reverse partial sequences of SXB19 xylanolytic bacterial isolate obtained from 16S rRNA gene sequencing are shown to retain a length of 882 and 856 bp, respectively (Fig. S1 and Fig. S2).

### Phylogenetic tree analysis of xylanolytic bacterial strain SXB19

**Fig. 4:**
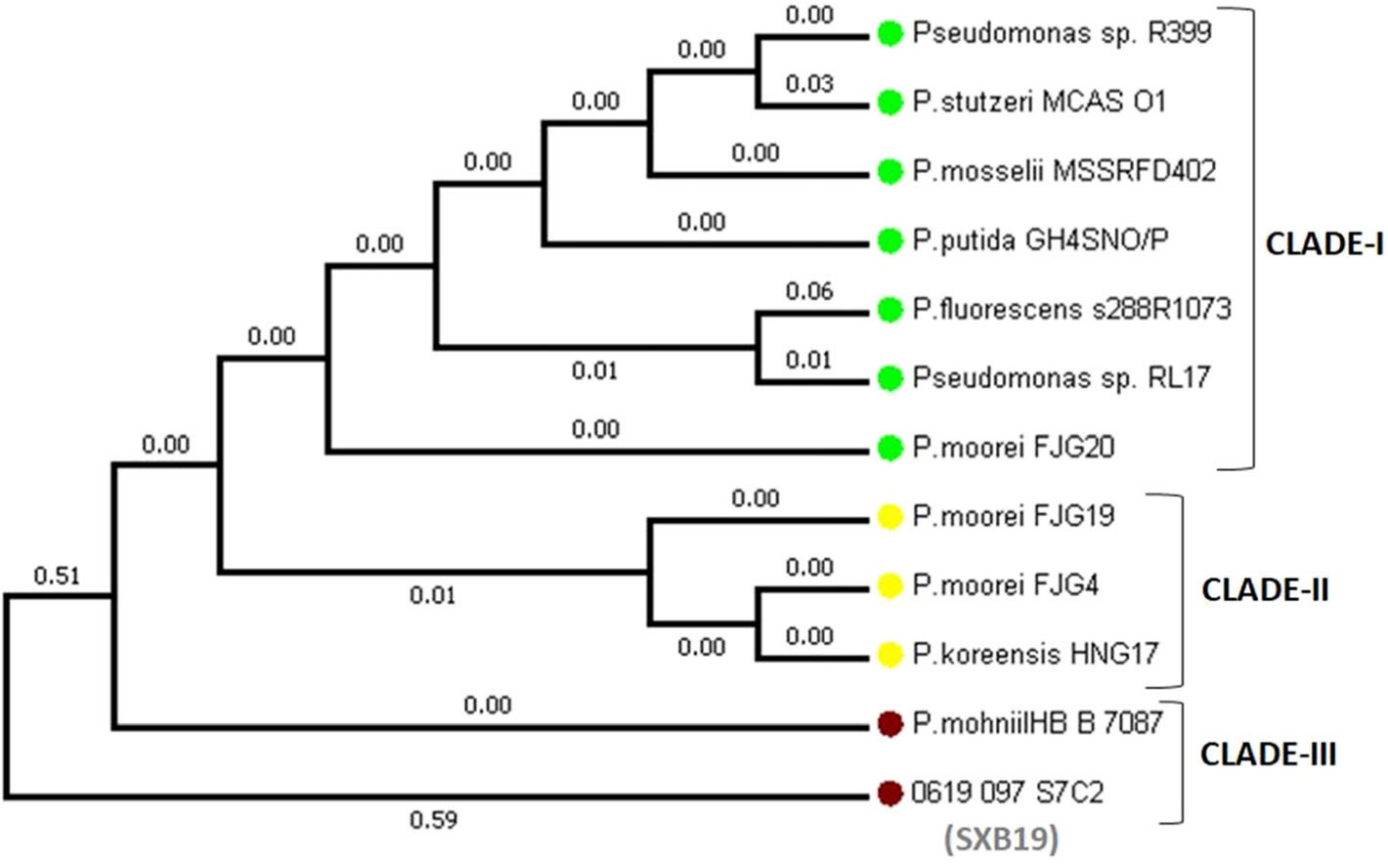
Phylogenetic tree for the bacterial isolate SXB19

The neighbor-joining method was performed to construct the phylogenetic tree using the 16SrRNA sequence data of SXB19 for the identification of the bacterium. Based on the results from the phylogenetic tree analysis, it can be observed that the 16S rRNA gene sequence alignment of isolate SXB19 is very closely related to *Pseudomonas mohnii* HB B 7087 with the highest similarity index of 99% under the CLADE-III (Fig. 4). Therefore, on the basis of 16S rRNA gene sequencing and phylogeny analysis, the SXB19 strain was identified as *Pseudomonas mohnii*.

### Optimization of xylanolytic enzyme activity

Different inorganic nutrients like carbon, nitrogen, and metal sources are indispensable for the optimum growth of bacteria and its enzyme activity. Hence, experiments were conducted to evaluate the effect of carbon, nitrogen, and metal ions on xylanase activity of *Pseudomonas mohnii*. Further, optimization of different parameters like pH, temperature, incubation time, and substrate concentration to maximize the xylanase activity of the bacterium *P. mohnii* was also performed using the response surface method.

### Effect of carbon sources on xylanase activity

**Fig. 5:**
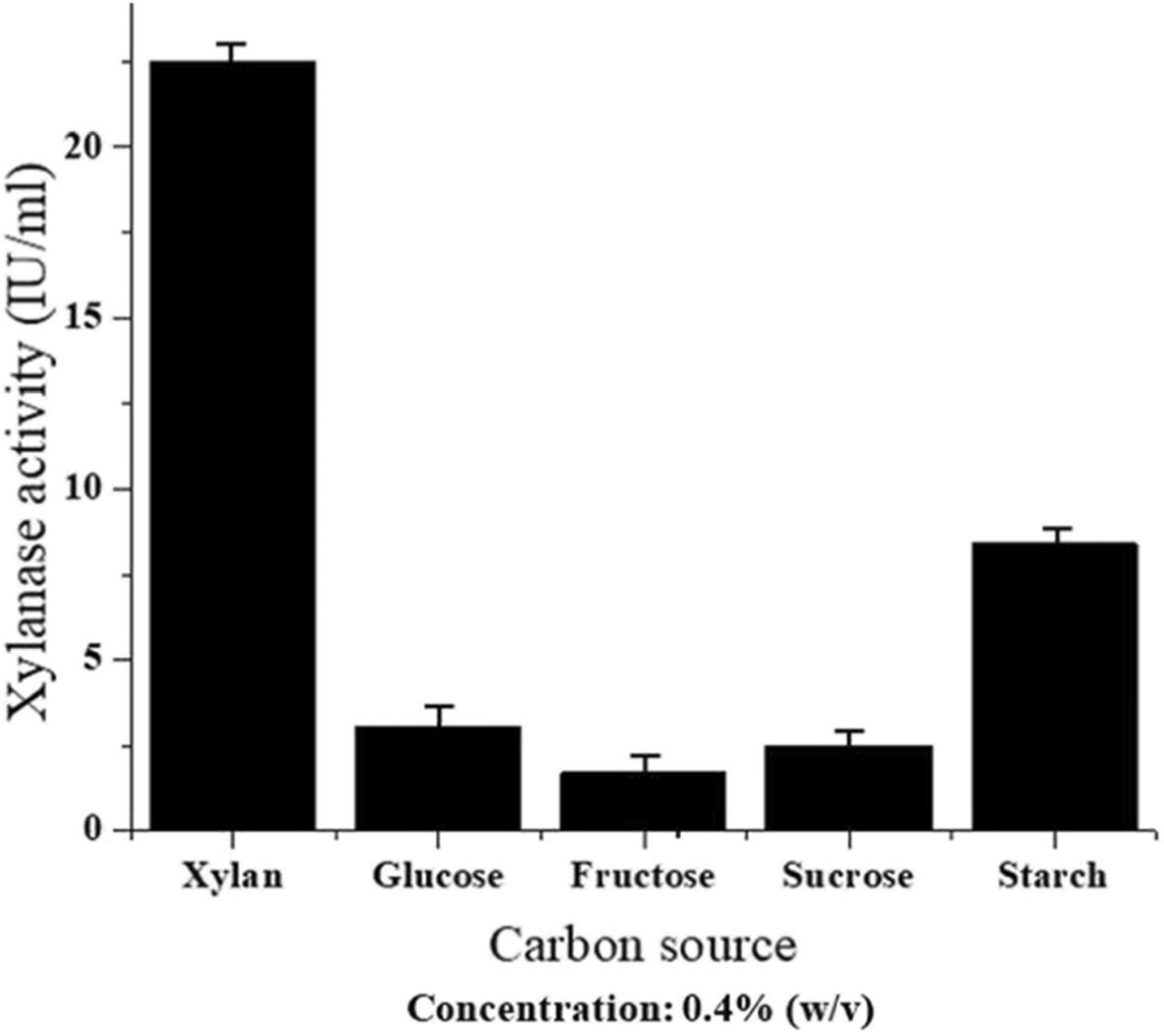
Effect of different carbon sources on xylanase activity of the bacterium *Pseudomonas mohnii*

The effect of different carbon sources on xylanase activity along with xylan was performed in the same basal media and culture conditions. When xylan was replaced with different carbon sources, viz. glucose, fructose, sucrose, and starch in the growth medium of the bacterium *Pseudomonas mohnii*, maximum xylanase-specific activity was recorded with xylan (22.5 IU/ml as calculated from enzyme determination), followed by starch (8.4 IU/ml), glucose (3.04 IU/ml), sucrose (2.5 IU/ml), and fructose (1.71 IU/ml) (Fig. 5). This result depicted that xylan compared to other carbon sources strongly induce xylanase production. A notable reduced activity was observed in the case of glucose, fructose, and sucrose, which may be resulted from the fact that easily metabolizable substrates inhibit the enzyme activity.

### Effect of nitrogen sources on xylanase activity

**Fig. 6:**
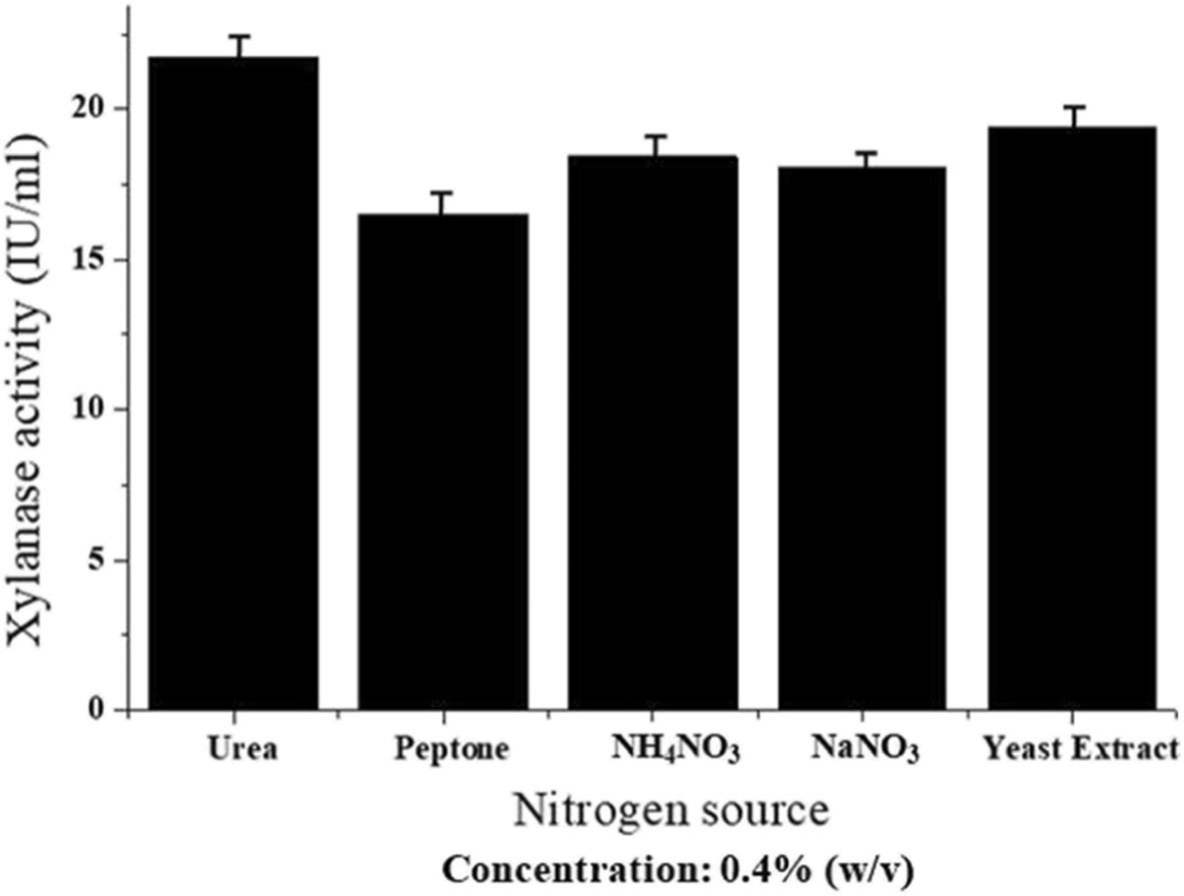
Effect of different nitrogen sources on xylanase activity of the bacterium *Pseudomonas mohnii*

Enzyme activity is also significantly regulated by one of the major factors which is a different nitrogen source. Maximum xylanase activity of 21.72 IU/ml for the strain of *Pseudomonas mohnii* was obtained with the growth medium supplied with 0.4% urea (Fig. 6). The second highest xylanase activity with a value of 19.39 IU/ml was observed for yeast extract followed by other three nitrogen sources viz. NH_4_NO_3_ (18.42 IU/ml), NaNO_3_ (18.09 IU/ml), and peptone (16.49 IU/ml) (Fig. 6).

### Effect of metal ion sources on xylanase activity

**Fig. 7:**
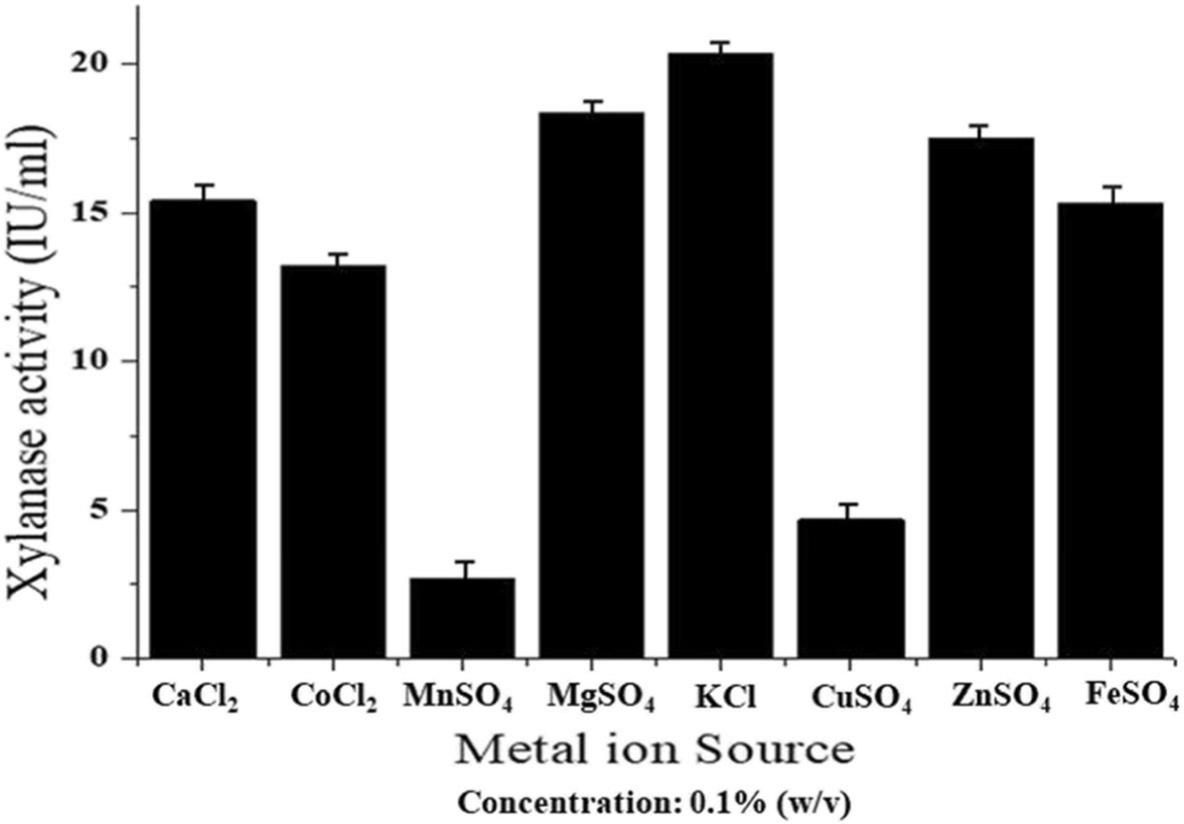
Effect of different metal ion sources on xylanase activity of the bacterium *Pseudomonas mohnii*

Xylanase activity was shown to greatly influenced by the metal ion source. The addition of metal ion sources such as MnSO_4_ and CuSO_4_ in the growth medium of *Pseudomonas mohnii* showed a significant decrease in the enzyme activity with a value of 2.71 IU/ml and 4.66 IU/ml, respectively (Fig. 7). Other selected metal ion sources viz. CaCl_2_, CoCl_2_, MgSO_4_, ZnSO_4_, and FeSO_4_ had not such notable impact on xylanase activity, while a slight stimulatory effect of the chemical additive KCl on xylanase activity (20.35 IU/ml) of *Pseudomonas mohnii* was observed (Fig. 7).

### Optimization of growth factors through response surface methodology

In this study, RSM was used to determine the xylanase activity for bacterial strain *Pseudomonas mohnii* in different growth factors. There are four independent variables such as incubation time, pH, substrate conc., and temperature based on which the responses were calculated. The full experimental plan as per the designed parameters along with the response values are given in Table 4.

**Table 4:** Optimization of fermentation parameters using response surface methodology with Box-Behnken design

SL. No.	Incubation (h)	pH	Xylan Conc. (%)	Temperature (°C)	Xylanase activity Experimental (IU/ml)	Xylanase activity Predicted (IU/ml)
1.	36	6.0	1	50.0	83.70	84.38
2.	36	9.0	1.5	35.0	149.90	145.08
3.	36	6.0	1.5	35.0	25.10	26.85
4.	60	7.5	1	50.0	48.70	51.04
5.	36	7.5	1.5	35.0	63.20	63.2
6.	60	7.5	1.5	50.0	52.60	55.57
7.	12	6.0	1	42.5	104.30	103.03
8.	12	7.5	0.5	42.5	65.70	67.56
9.	60	6.0	1	42.5	84.00	83.10
10.	60	7.5	0.5	50.0	148.90	146.09
11.	36	7.5	1	42.5	54.20	55.57
12.	60	7.5	0.5	42.5	147.8	146.08
13.	60	9.0	1	35.0	149.00	149.41
14.	60	7.5	1	35.0	97.80	96.65
15.	12	7.5	1	35.0	60.08	58.52
16.	36	9.0	1	50.0	68.00	66.97
17.	36	6.0	1	35.0	75.40	77.53
18.	36	7.5	0.5	35.0	105.10	103.34
19.	12	9.0	1	42.5	81.00	80.02
20.	60	9.0	1.5	42.5	141.20	140.59
21.	12	7.5	1	50.0	56.60	58.53
22.	36	7.5	0.5	35.0	59.90	59.13
23.	12	7.5	1.5	42.5	87.00	87.01
24.	36	7.5	1.5	50.0	56.90	56.79
25.	12	9.0	0.5	42.5	40.80	42.83
26.	36	6.0	0.5	42.5	152.00	152.60
27	36	7.5	1	42.5	54.20	55.57

### Regression analysis

A regression analysis was performed to determine the optimum conditions that result in the maximum xylanase activity. Using multiple regression analyses, a second-order polynomial equation was developed which represents the relationship between enzyme activity, incubation time, pH, substrate concentration, and temperature. The fitted response of the model is calculated as follows:

$$ \mathrm{Xylanase}\ \mathrm{activity}\ (Y)=+55.57+7.66\times \mathrm{A}+6.12\times \mathrm{B}-11.88\times \mathrm{C}-11.40\times \mathrm{D}+17.62\times \mathrm{A}\mathrm{B}-21.60\times \mathrm{A}\mathrm{C}-11.40\times \mathrm{A}\mathrm{D}+41.00\times \mathrm{B}\mathrm{C}-14.82\times \mathrm{B}\mathrm{D}+7.23\times \mathrm{C}\mathrm{D}+11.36\times {\mathrm{A}}^2+32.25\times {\mathrm{B}}^2+18.02\times {\mathrm{C}}^2-0.7475\times {\mathrm{D}}^2 $$

In the above equation, *Y* is the response value of xylanase activity. Incubation time, pH, substrate concentration, and temperature were represented by A, B, C, and D, respectively. The significance of the second-order polynomial equation for xylanase activity was measured by the analysis of variance (ANOVA). The coded equation assists to determine the relative impact of the factors by analyzing the factor coefficient. The polynomial equation implied that for the expression of xylanase activity, the parameters like A, B, C, D, AB, AC, AD, BC, BD, CD, A^2^, B^2^, and C^2^ are the most significant terms of the experimental model. The coefficient of variation (CV) denotes the degree of precision with which the experimental conditions are compared. In the present study, a CV value of 3.22% has been recorded for the RSM model of xylanase activity for *Pseudomonas mohnii*. Calculated *p* value as obtained from the coefficient table was recorded as < 0.0001 which signifies the major impact of A, B, C, D, AB, AC, AD, BC, BD, CD, A^2^, B^2^, and C^2^ towards the highest coefficient value for xylanase activity.

### Statistical analysis

To check the statistical significance of the quadratic model developed for xylanase activity of *Pseudomonas mohnii*, RSM was used to perform the *F* test and the analysis of variance (ANOVA). It has been shown from the obtained result that the model was highly significant, as suggested by the calculated *F* value (230.17) and low probability value (< 0.0001). The lack of fit *F* value of 0.3782 indicates that this value is not significant to the pure error. There is an 88.03% chance that a lack of fit *F* value goes larger due to noise (Table 5). This non-significant lack of fit as represented from the ANOVA table evidence the optimization of the model system. The coefficient of determination (*R*^2^) was calculated as 0.9963 for enhanced xylanase activity, representing that the statistical model can explain the adequate variability in the response. Similarly, The predicted *R*^2^ value of 0.9831 was in well agreement with the adjusted *R*^2^ value of 0.9920. This represents a good coherence between the experimental and predicted values for xylanase activity. If there are many terms in the model and the sample size is not very large, the adjusted R^2^ value may be noticeably smaller than the R^2^. In the present study, in concur with the above statement, adjusted *R*^2^ 0.9920 is also less than the *R*^2^ value 0.9963 (Table 6).

**Table 5:** Analysis of variance (ANOVA) for the quadratic model of xylanase enzyme activity as per Box-Behnken design

Source	Sum of Squares	Degree of freedom	Mean Square	F-value	***p***-value
Model	22,859.79	14	1632.84	230.17	< 0.0001
A-Incubation time	704.11	1	704.11	99.25	< 0.0001
B-pH	448.96	1	448.96	63.29	< 0.0001
C-Substrate Conc.	1692.19	1	1692.19	238.53	< 0.0001
D-Temperature	1559.06	1	1559.06	219.77	< 0.0001
AB	1242.56	1	1242.56	175.15	< 0.0001
AC	1866.24	1	1866.24	263.07	< 0.0001
AD	520.30	1	520.30	73.34	< 0.0001
BC	6724.00	1	6724.00	947.82	< 0.0001
BD	879.12	1	879.12	123.92	< 0.0001
CD	208.80	1	208.80	29.43	0.0002
A^2^	688.87	1	688.87	97.10	< 0.0001
B^2^	5548.72	1	5548.72	782.15	< 0.0001
C^2^	1731.36	1	1731.36	244.05	< 0.0001
D^2^	2.98	1	2.98	0.4201	0.5291
Residual	85.13	12	7.09		
Lack of fit	55.68	10	5.57	0.3782	0.8803
Pure Error	29.45	2	14.72		
Cor Total	22,944.92	26

**Table 6:** Fit statistics of the response model for xylanase activity of *Pseudomonas mohnii* calculated by response surface methodology

R^**2**^	Adjusted R^**2**^	Predicted R^**2**^	Adeq Precision	C.V. %	Standard deviation
0.9963	0.9920	0.9831	53.2680	3.22	2.66

### Interaction among variables

The effects of interactions among the variables on xylanase activity were studied. Interactive effects of any two variables were represented by optimized 3D surface plots while keeping the other variable fixed at the point values of different levels.

**Fig. 8:**
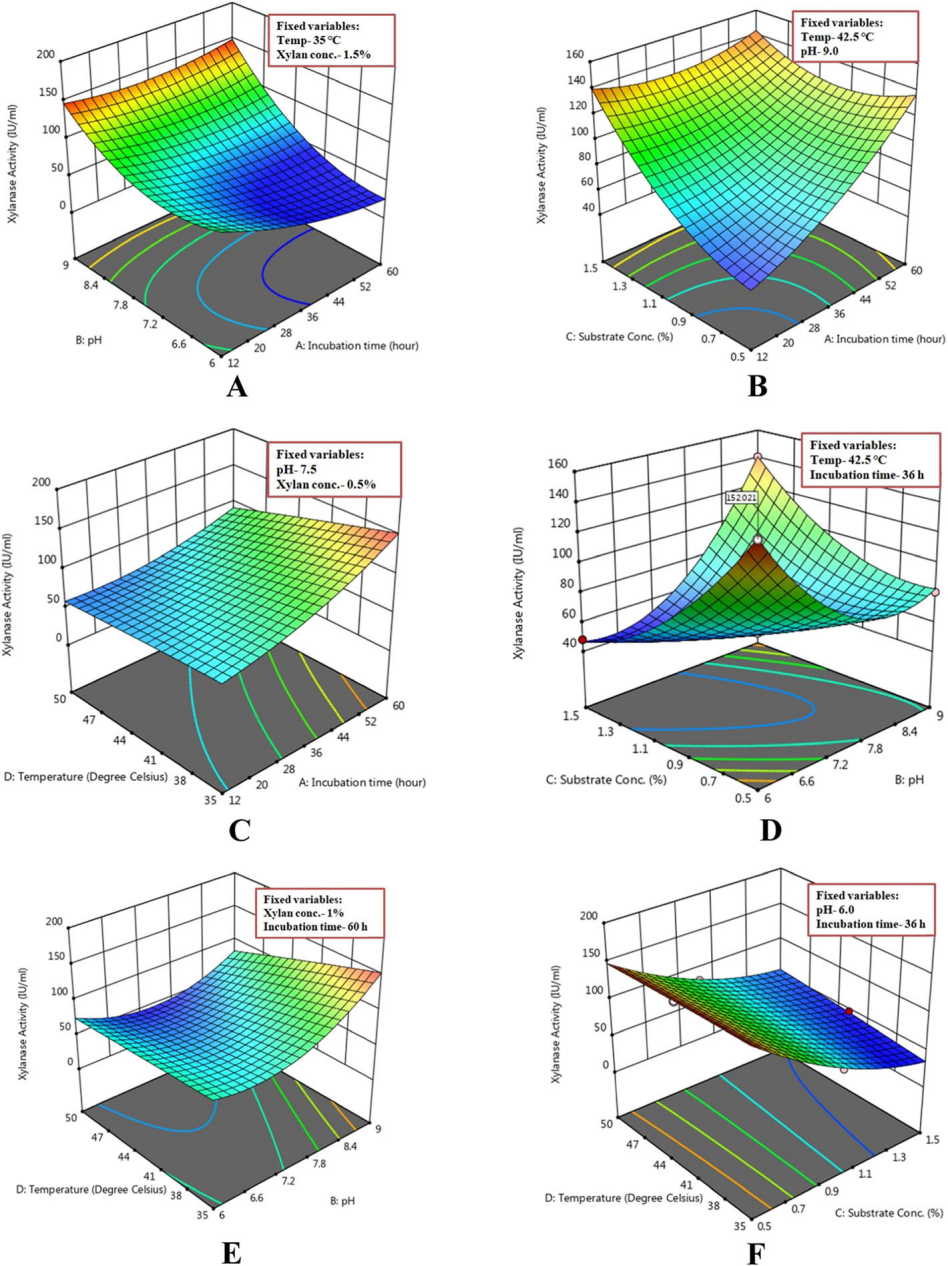
Response surface graph of xylanase activity of the xylanolytic bacterium *Pseudomonas mohnii* with respect to different experimental factors **a** vs. pH, incubation time; **b** vs. substrate conc., incubation time; **c** vs. temperature, incubation time; **d** vs. substrate conc., pH; **e** vs. temperature, pH; and **f** vs. temperature, substrate conc.

**Fig. 9:**
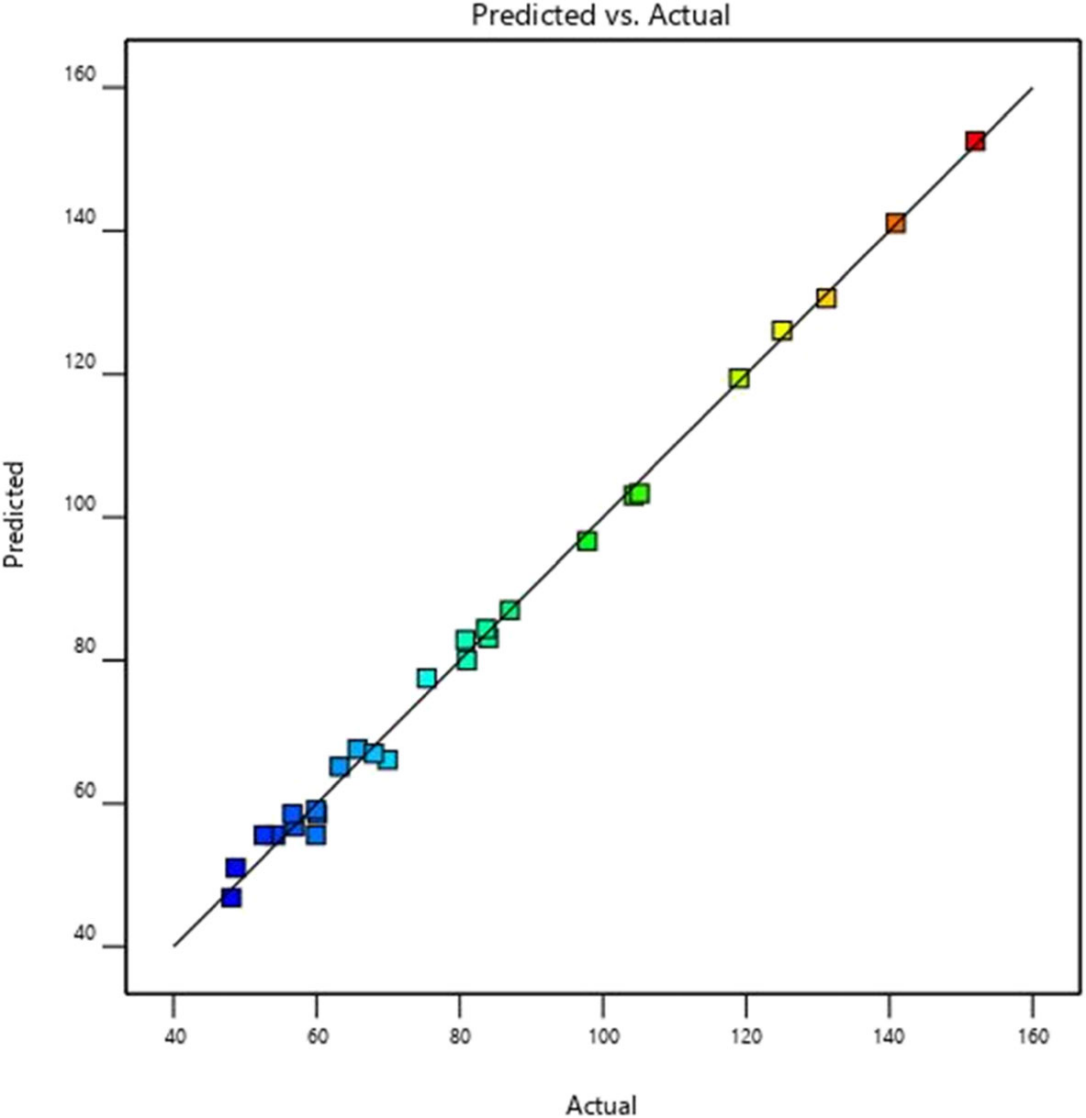
Residual plots for the xylanase activity of the xylanolytic bacterial strain *Pseudomonas mohnii* calculated from RSM for the validation of predicted and actual xylanase activity

The response surface curves represented how xylanase production was a function of pH and incubation time by keeping the levels of temperature and substrate conc. constant at 35 °C and 1.5%, respectively. The increase in pH in the same time resulted in the elevation of xylanase activity from 25.10 to 149.9 IU/ml (Fig. 8a). The response surface plots represented in Fig. 8b show that there was a high production of xylanase (141.2 IU/ml) at substrate conc. 1.5% and incubation time 60 h and low yields (40.8 IU/ml) at substrate conc. 0.5 and incubation time 12 h with constant temperature and pH. Figure 8c presents the response surface curves which revealed how xylanase production was a function of temperature and incubation time by keeping the levels of pH and substrate conc. constant at 7.5 and 0.5%, respectively. The increase in incubation time and temperature resulted in an increment of xylanase activity from 59.9 to 147.8 IU/ml. The response surface curves as presented in Fig. 8d depict how xylanase production was a function of substrate conc. and pH by keeping the levels of temperature and incubation time constant at 42.5 °C and 36 h, respectively. In this condition, an increase in substrate conc. resulted in an increase of xylanase activity from 48.10 to 152 IU/ml. The response surface curves depicted in Fig. 8e show that there was high production of xylanase (149 IU/ml) at temperature 35 °C and pH 9.0 but low yields (52.60 IU/ml) at a higher temperature of 50 °C and a lower pH of 7.5 while other two variables substrate conc. and incubation time were kept fixed at 1% and 60 h, respectively. Figure 8f represents the response surface curves which depicted how xylanase production was a function of substrate conc. and temperature by keeping the levels of incubation time and pH constant at 36 h and 6, respectively. In this condition, the result showed that a decrease in substrate conc. from 1.5 to 0.5% resulted in promoting the xylanase activity from 52.6 to 148.9 IU/ml. The residual plot (Fig. 9) analysis of xylanase activity of the xylanolytic bacterial strain *Pseudomonas mohnii* represented greater stability in the predicted vs. actual plots and normal residual plots. The distribution of response data from different experimental conditions depicted the equal contribution of most of the factors. The probability plots also demonstrated as much similarity between the predicted and actual xylanase activity.

In the present study, an optimum pH 6.0 was determined in which the highest amount of xylanase (152 IU/ml) was produced. Although at pH 9.0, 149.9 IU/ml of xylanase was shown to be produced. In the present study, the production of xylanase was reported for different incubation time periods such as 12, 36, and 60 h. The optimum incubation time and substrate concentration were 36 h and 0.5%, respectively, upon which the bacterial strain *Pseudomonas mohnii* showed the highest xylanase activity. The role of temperatures on xylanase enzyme production was determined in the current study, and the results showed maximum enzyme production takes place at 42.5 °C by the bacterium *Pseudomonas mohnii*.

Among the different bacterial species studied, a great number of species belong to the genus *Bacillus* are found to exhibit xylanase activity. However, a review of the literature revealed that a relatively smaller number of species that belongs to the *Pseudomonas* genus has been reported to show xylanase activity. A comparison of xylanase activity from different species from *Pseudomonas* such as *Pseudomonas boreopolis*, *Pseudomonas cellulosa*, and *Pseudomonas* sp. WLUN024 is been presented in Table 7 along with the strain of *Pseudomonas mohnii* bacterium studied in the present work [35–38]. Among the different *Pseudomonas* strains, *Pseudomonas* sp. WLUN024 exhibited so far the maximum xylanase activity 190.2 IU/ml followed by *Pseudomonas mohnii* (152 IU/ml), *Pseudomonas boreopolis* (25.61 IU/ml), *Pseudomonas boreopolis* G-22 (1.75–2.67 IU/ml), and *Pseudomonas cellulosa* (0.73 IU/ml). From these values of xylanase activity, it is apparent that the strain *Pseudomonas mohnii* is an efficient xylanase-producing bacterium that belongs to a new report which has not been studied before.

**Table 7:** Comparison of xylanase production by different *Pseudomonas* sp.

Bacteria	Xylanase activity (IU/ml)	Feedstock	Incubation time/Temperature	pH	Reference
*Pseudomonas boreopolis*	25.61	Wheat bran	96 h, 65 °C	6.0	[35]
*Pseudomonas boreopolis* G22	1.75–2.67	Wheat straw	5 days	8.8	[36]
*Pseudomonas cellulosa*	0.73 ± 0.07	Oat spelt xylan	30 h	7.0	[37]
	0.65 ± 0.07	Glucuronoxylan			
	0.81 ± 0.10	Rye arabinoxylan			
	0.91 ± 0.11	Wheat arabinoxylan			
	0.21 ± 0.02	β-Glucan			
	<0.01	Carbo galactomannan			
	<0.01	Pectin			
	<0.01	LB			
	0.21 ± 0.04	LB and oat spelt xylan			
	<0.01	Oat spelt xylan/glucose			
*Pseudomonas* sp. WLUN024	118.7 ± 5.8	Hemicellulose	24 h, 37 °C	7.2–8	[38]
	190.2 ± 4.3	Xylan (Sigma)			
	32.2 ± 1.2	Xylose			
	31.2 ± 1.0	Wheat bran			
	1.2 ± 0.3	Starch			
	0.5 ± 0.3	Cellobiose			
	1.4 ± 0.4	Sucrose			
	2.0 ± 0.3	Glucose			
*Pseudomonas mohnii*	152.0	Corncob xylan	36 h, 42.5 °C	6.0	[Present study]

## Discussion

Isolation and screening of bacterial strains are a crucial and principal step for the further study of their identification, secreted enzyme assay, and other molecular and biochemical characterizations. In the present study, xylanolytic bacteria were isolated from the forest soil of Simlipal Biosphere Reserve and screened for their xylanase activity using the corn cob xylan. There are different sources of xylans apart from corn cob such as corn fiber, birchwood which are also generally used to isolate and screen xylonolytic bacteria [14]. Similar to our study, isolation and screening of xylanolytic bacteria from the soil samples using a corn cob xylan medium have also been reported by Alves-Prado et al. [39]. It is apparent from the present study that forest soil samples are a reservoir for a large number of xylanase-producing bacteria. It is known that soil is the common source for isolating various industrially important microorganisms [40]. This is also evidenced that forest soil contains organic constituents and minerals which make it a high microbial diversity hub [41]. Earlier studies also indicated that bacterial diversity is abundant in forest topsoils as they are rich in decomposing litter materials [42].

In the current study, xylanase activity has been determined using the DNS method. Similar to this study, Ho [43] also used the DNS method for the estimation of xylanase activity of the bacterium *Bacillus subtilis* ATCC 6633. Interestingly, the xylanase activity of the bacterium, *Bacillus subtilis* ATCC 6633 recorded by Ho [43] was 22.07 IU/ml which is very close with the xylanase activity (22.5 IU/ml) as obtained from the most potent xylanolytic strain in the present investigation.

The comparison of growth of the bacterium SXB19 in the present study with the results of previous literatures revealed that exponential phase of SXB19 started faster than some already studied *Bacillus* sp. in which exponential growth started after the incubation of 24 h [44]. Yadav et al. [45] in their study showed that the exponential growth of *Anoxybacillus kamchatkensis* NASTPD13 started after 6 h of incubation and ended at 26 h. These temporal variations in bacterial exponential growth might be resulted from different culture conditions and the amount of bacterial inoculums in the culture broth which has also been demonstrated in the study conducted by Emami et al. [37] where the same carbon source xylan was used the same as the present study. The determination of the exponential phase is crucial to ensure the cells are active. There is an increased enzyme activity in respect to the SXB19 growth phase was recorded which suggested that the xylan was actively used by SXB19 strain during this phase [45]. The growth curve of SXB19 as determined in the present study showed that the stationary phase was delayed that of previously reported result for *Bacillus licheniformis* 7-2 which started its stationary phase after 24 h [46]. Similar to our study, the xylanase production by *Geobacillus stearothermophilus* KIBGE-IB29 represented a rapid increase after 24 h [47]. Xylanase activity of SXB19 becomes significantly declined during the dye off phase. This decrease in xylanase activity might be resulted from the generation of toxic metabolites during bacterial growth which prevents the enzyme production [48]. Another cause of the decreased enzyme activity might be the feedback inhibition resulted from the high yield of xylose produced from xylan degradation [47]. The reduced enzyme activity at the end of the bacterial growth phase could also be caused by intracellular protease action [49] or may be due to the abrupt changes in the pH of the media [50].

In the present study, the 16S rRNA sequencing method was used for the molecular identification of bacterial strains [51]. Some bacteria from the genus *Pseudomonas* having xylanolytic activity over a wide range of environmental conditions have already been reported [22, 35–38]. There was a previous report that the strain *Pseudomonas mohnii* is also capable of degrading a toxic substance like cholorosalicylates or isopiramic acid, an effluent from paper mill [52, 53]. However, the present finding of xylanase from the bacterium *Pseudomonas mohnii* is a new report which has not been studied earlier.

In the present investigation, it has been shown that xylanase activity of *Pseudomonas mohnii* becomes significantly decreased in the presence of glucose in the growth medium of the bacterium. Rojas-Rejo´n et al. [54] in their study also demonstrated the inhibitory effect of glucose on xylanase activity. The result from their study showed that when 20 mM glucose was added, the xylanolytic activity of the bacterium *Cellulomonas flavigena* PN-120 decreased by 41% compared to the culture grown without glucose [54]. So, from the observation of the present study and the work reported from the previous study of Rojas-Rejo´n et al., it can be stated that the activity of xylanase enzyme is not solely regulated by the substrate xylan and can also be retained or downregulated to a certain level in the presence of different other carbon sources [54].

In the case of nitrogen source, urea has been determined to have the most inducing effects in increasing the xylanase activity of *Pseudomonas mohnii* among all tested nitrogen sources. In a similar type of study conducted by Seyis and Aksoz [55], it has also been reported that urea is a major inducer in the aspect of increasing the xylanase activity most as an additional nitrogen source compared to other sources like NH_4_NO_3_, NaNO_3_, and (NH_4_)_2_SO_4_. Kumar et al. [56] demonstrated in their study that a low concentration of urea can induce structural changes which enhance the activity of xylanase enzyme from the bacterial species of *Chinia*. Thus, it can be stated from the result obtained from the present study and the previous reports that the presence of urea in the bacterial growth media might have some inducing effect to promote xylanase activity.

In the present study, a strong inhibition on xylanase activity of *Pseudomonas mohnii* by the metal ion sources MnSO_4_ and CuSO_4_ has been recorded. Inhibitory effect by these two chemical additives on xylanase activity of *Bacillus* species was also shown in a previous study by Mamo et al. [57]. KCl has no significant effect on xylanase activity as recorded in this present study. This observation is similar to the result obtained from the investigation by Archana and Satyanarayana [58] on xylanase production by a thermophilic bacterium *Bacillus licheniformis* A99.

Optimization of growth factors of the bacterium *Pseudomonas mohnii* for maximizing the xylanase activity was performed by RSM. The significance of the experimental model designed in RSM is validated by the coefficient of variation (CV). A high percentage of CV represents the lower reliability of any such experimental model [59]. Therefore, in the present study, a lower CV value of 3.22% implies that the experiments performed are highly reliable. A model can be considered practically reliable and highly significant if the CV is less than 10% [60].

Doddapaneni et al. [60] suggested that in an ANOVA experiment, the closer the value of *R*^2^ to 1.0, the stronger the model and the better it predicts the response of tested enzyme activity according to the designed experimental conditions. If there are many terms in the model and the sample size is small, the adjusted *R*^2^ value should be significantly less than the *R*^2^ [61]. In the current study, we have recorded a less adjusted *R*^2^ value than that of *R*^2^. Based on the results from ANOVA obtained from the present study, it can be stated that the designed experimental model is highly significant and correctly represents the actual relationship of the effects of variables and enzyme response related to xylanase activity.

Enzyme activity depends significantly on various parameters like pH, substrate concentration, and temperature. The pH is one of the major factors responsible for the functioning of enzyme systems within an organism [62]. RSM results of the xylanase activity from *Pseudomonas mohnii* showed that the enzyme has a high substrate turnover rate at pH 6.0 and 9.0. The observations from the result of RSM suggested that xylanase production at different pH may be an indication that the bacterium *Pseudomonas mohnii* may have the efficiency to induce multiple xylanase activity at different pH [63]. Similar to this study, Sá-Pereira et al. [64] reported a *Bacillus* strain, *Bacillus subtilis*, which showed maximum xylanase activity at pH 6.0. Xylanase from *B. licheniformis* A99 and *B. coagulans* BL69 was reported to have the optimum pH of 7 for their maximum enzyme activity [58, 65]. Gupta et al. [66] reported that purified xylanase was functional between pH 6.0 and 10.5 and able to retain more than 70% of its functionality in this pH range.

In the current study, an increase in xylanase activity (52.60 to 148.90 U/ml) of *Pseudomonas mohnii* has been recorded at 50 °C with a decrease in substrate conc. from 1.5 to 0.5. Likewise, Shanthi and Roymon [40] in their study have also shown the highest xylanase production at 50 °C. In another study, Shakoori et al. [67] reported a maximum xylanase production at 45 and 50 °C. Irfan et al. [48] in their study reported that the optimum temperature of xylanase activity was 50 °C and pH were 5 and 5.5 by *B. subtilis* BS04 and *B. megaterium* BM07, respectively.

## Conclusion

In the present study, a potent xylanase-producing bacterium SXB19 was isolated from the forest soil of Simlipal Biosphere Reserve, Odisha, India, and was identified as *Pseudomonas mohnii* based on phenotypic and 16S rRNA sequencing. Xylanase production of this bacterium was optimized by the response surface methodology using Box-Behnken for the determination of growth parameters (pH, temperature, substrate concentration, and incubation time) in enhancing the xylanase production of the isolated bacterium. The optimization of the xylanolytic activity revealed a higher xylanase-producing activity (152 IU/ml) by *Pseudomonas mohnii* at an optimized condition of incubation time 36 h, pH 6.0, xylan concentration 0.5%, and temperature 42.5 °C. The enzyme production showed an approximately 6.75 times increase over the unoptimized condition. Apart from xylan, starch as a carbon source, urea as a nitrogen source, and metal ion sources like KCl and MgSO_4_ also reported to have a significant impact on the xylanase activity of this bacterium. Overall, the bacterium reported in this study was considered to be a potential source of xylanase which can be exploited for industrial application including bioethanol production; however, further studies are required in this direction.

## Supplementary Information

**Additional file 1: Fig. S1** Forward 16S rRNA gene sequence of strain SXB19. **Fig. S2** Reverse 16S rRNA gene sequence of strain SXB19.

## Data Availability

All data generated or analyzed during this study are included in this article.

## Abbreviations

SBR: Simlipal Biosphere Reserve
SXB: Simlipal xylanolytic bacteria
RSM: Response surface methodology
KCl: Potassium chloride
MgSO_4_: Magnesium sulfate
MnSO_4_: Manganese sulfate
ZnSO_4_: Zinc sulfate
CuSO_4_: Copper sulfate
FeSO_4_: Ferrous sulfate
NaCl: Sodium chloride
CaCl_2_: Calcium chloride
CoCl_2_: Cobalt chloride
NaNO_3_: Sodium nitrate
NH_4_NO_3_: Ammonium nitrate
(NH_4_)_2_SO_4_: Ammonium sulfate
IEA: International Energy Agency
DNS: 3,5-Dinitrosalicylic acid
