# Supplementary material for: Optimization of xylanase from Pseudomonas mohnii isolated from Simlipal Biosphere Reserve, Odisha, using response surface methodology
Source: J Genet Eng Biotechnol. 2020 Dec 11;18:81. doi: 10.1186/s43141-020-00099-7 (PMC7732945; doi:10.1186/s43141-020-00099-7)
Supplement: Supplementary file 1 — Additional file 1: Fig. S1 Forward 16S rRNA gene sequence of strain SXB19. Fig. S2 Reverse 16S rRNA gene sequence of strain SXB19. [file 43141_2020_99_MOESM1_ESM.docx]

**>0619_097___S7C2_785F_G09.ab1**

TCTGAGTAGGCGCAGCTAACGCATTAACTTTGACCGGCCTGGGGGAGGAGGCAGGGCAGGGTTAAAACTCAAAGGAATTGACGGGGGCCCGCACAAGGGGGGAAGCAGGTGGTTTAATTCAAAGCAACGCGAAAAACCTTACCAGGCCTTGACATGCAGAGAACTTTCCTGAAATGGATTGGTGCCTTCGGGAACTCTGACACAGGTGCTGCATGGTTGTCGTCAGCTCGTGTCGTGAGATGTTGGTTTAATTCCCGCAACGAGCGCAACCCTTGTCCTTATTTACCACCACGTTATGGTGGGCACTCTAAGGAGACTGCCGGTGACAAACCGGAGGAAGGTGGGGATGACGTCAAGTCATCATGTCCCTTACGGCCTGGGCTACACACGTGCTACAATGGTCGGTACAAAGGGTTGCCAAGCCCCGAGGTGAACCTAATCTCACAAAACCAATCGTATTCCGGATCGCACTCTGCAACTCCACTGCGTGAATTCGGAATCCCTAGTAATCCCCAATCGTAGGGGCGCGCCCAATACATTCCCGGTGCTTGTAAACCCCGCCGCTCTCCCCCCCGGGGGGTGTTTTTACGGAGTTAGCTGGCCCCACCTTCTGGAGGAGGGTCACCACGGGTGTGATTCTTTCAGTGGGGGGGGGGCACTAACAAGGGTAACCTATATCTGTTTGGCCCCCCAGCTTTTCCCCCCTCCAGGGCCAGAAAGGAACCAAGGAGGCCCCCCTTTCCCCCCGGGGGGTCCCTTCCAAATATTCACCAAATTTCCCCGCCCCCCCGAGAAATTTCCCCCCCCCCTTCTCCGAACCTAAAACCCCCGTTTTGGAGCCCGTCTCCCCAAGTGAGAAACCCGGGGTTTTTCACCCCCGGA

**Fig. S1** Forward 16S rRNA gene sequence of strain SXB19.

**>0619_097___S7C2_907R_D01.ab1**

TTAGCTGCGCCACCGAACACTCTAGGATTCGACGGCTAGATGACTCCGTTTACGGGGGGAACAACCAGGGTACCTAACCTGGTTGGTCCCCCAGCCTTTCCCCCCCCGCGGCCAGTATGGACCCAGGAGGCCCCCTTCCCCCTGGGGGTCCTTCCATATTCAACAATTTTCCCCCCTACCCCGGAATTTCCCCTACCCCCTCCCGAACCCAAACTCGCCATATTTGAATGAATTTCCCAGGTTAACTCTGGGACTTCACCCCCGAATTTAACAAACCCCCAAGTGGCGCTTTACCCCCATAAATTCCAATAACCGTTTGCCCCTTCTGAATAACCCCGCTGGCTGCCCCAAATTTAGCCGGTCCTCATTCTCCACTAAACGCCATAACATCATCGTATAAACTTATTTCCCTTCCTCCCGCCTTAAACAGCTTTACAGCCCGAAAACCTTCTTCACACGCCCGGCGTGGGGAGAGTCTCCCCTGCTCCCTTCGAAAAAAATTCCCGCCTGCGTCCCTTGGGTAGGGGCCCCGGACCATTCCTTTGATCCATATTCTGGGGGCCCCCCCTCCCCAAGGCGTTACGCTTAATGCTTTAGTTGCCCCCTTAAACCCCTAACGGGCCCGAACGGACATATGCCCTCCTTACGGGGGGGGGGACTACAAGGGAATTTACTTCTCGTTTTGCCCCCCCACGTTTTTCAAACCCCGTCGTTTTAAAAAGACCAAGGTGCCCCTCCTCCGCCAATGCGGTCCCTTTAAAATATCAACACGTTGCCGCAGCCAACGAGAAATTTCCCCCCCCTCCTATCGAATTCAAGATTCCCGGGTTTTGAAAGGGTTTCCCAGGTTAAACCT

**Fig. S2** Reverse 16S rRNA gene sequence of strain SXB19.
